# Time course of adverse reactions following BNT162b2 vaccination in healthy and allergic disease individuals aged 5–11 years and comparison with individuals aged 12–15 years: an observational and historical cohort study

**DOI:** 10.1007/s00431-022-04643-0

**Published:** 2022-10-13

**Authors:** Makoto Yoshida, Yurie Kobashi, Yuzo Shimazu, Hiroaki Saito, Chika Yamamoto, Takeshi Kawamura, Masatoshi Wakui, Kenzo Takahashi, Naomi Ito, Yoshitaka Nishikawa, Tianchen Zhao, Masaharu Tsubokura

**Affiliations:** 1 Faculty of Medicine, Teikyo University School of Medicine, Itabashi-ku, Tokyo, Japan; 2 Department of Internal Medicine, Serireikai Group Hirata Central Hospital, Ishikawa District, Fukushima, Japan; 3 Department of Radiation Health Management, Fukushima Medical University School of Medicine, Fukushima City, Fukushima, Japan; 4 Isotope Science Centre, The University of Tokyo, Bunkyo-ku, Tokyo, Japan; 5 Laboratory for Systems Biology and Medicine, Research Centre for Advanced Science and Technology (RCAST), University of Tokyo, Meguro-ku, Tokyo, Japan; 6 Department of Laboratory Medicine, Keio University School of Medicine, Shinjuku-ku, Tokyo, Japan; 7 Teikyo University Graduate School of Public Health, Itabashi-ku, Tokyo, Japan; 8 Research Center for Community Health, Minamisoma Municipal General Hospital, Minamisoma, Fukushima Japan; 9 Department of Pediatrics, Jyoban Hospital, Iwaki Fukushima, Japan

**Keywords:** Children, Coronavirus disease 2019, BNT162b2, Adverse reactions, Allergy, Asthma

## Abstract

We aimed to investigate the type and frequency of adverse events over 7 days following the first and second BNT162b2 vaccination. This observational and historical cohort study included patients aged 5–11 years who received two doses of BNT162b2 and provided consent along with their guardians. We collected data on sex, age, height, weight, blood type, history of Bacille Calmette-Guerin vaccination, allergic disease, medication, history of coronavirus disease 2019 (COVID-19), and adverse reactions 7 days following the first and second BNT162b2 vaccination using a questionnaire. Our results were compared with previously reported results for individuals aged 12–15 years. A total of 421 participants were eligible for this study. Among the 216 patients with allergic disease, 48 (22.2%) had experienced worsening of their chronic diseases, and the frequency of fatigue and dizziness after the second dose was higher than that of healthy individuals. The experience of systemic adverse reactions was associated with asthma. The frequency of headache, diarrhea, fatigue, muscle/joint pain, and fever after the second BNT162b2 vaccination was lower in individuals aged 5–11 years than in those aged 12–15 years. Fever was the only systemic adverse reaction that lasted longer than 5 days (1.0% of participants).

*Conclusions*: Individuals with allergic diseases, who are potentially susceptible to COVID-19, may experience worsening of their chronic diseases and more frequent adverse reactions after BNT162b2 vaccination than healthy individuals. To ensure that children with allergic diseases receive the vaccine safely, further information needs to be collected.

**Table:** 

**What is Known:** *• Adverse reactions after BNT162b2 vaccination among individuals aged 5–11 years are generally nonserious, more common after second vaccination, and substantially less common compared to those observed among individuals aged 12–15 years.*	
**What is New:** *• Individuals with allergic diseases experienced worsening of their chronic diseases and more frequent adverse reactions after BNT162b2 vaccination than healthy individuals.* • *Systemic adverse reactions were associated with asthma. Fever was the only systemic adverse reaction that lasted longer than 5 days.*

**Supplementary Information:**

The online version contains supplementary material available at 10.1007/s00431-022-04643-0.

## Introduction

COVID-19 was first confirmed in China in December 2019 and spread rapidly worldwide. This disease causes pneumonia and other respiratory diseases and has resulted in 6.34 million cumulative deaths as of July 5, 2022 [1]. COVID-19 also exerts an enormous impact on children. Although clinical manifestations of pediatric COVID-19 cases are generally less severe than those of adult cases [2–5], some children suffer from longer-term sequelae, such as multisystem inflammatory syndrome [4, 6,7,8] and long COVID [9]. Furthermore, social isolation measures, such as school closures and curfews associated with the COVID-19 pandemic have had a significant impact on children’s education [10–12], physical health, and mental health [13–17]. Therefore, safe and effective vaccines to prevent COVID-19 in children could dramatically reduce not only the physical effects of infection but also the marked social impact of the pandemic [18]. On December 11, 2020, the Food and Drug Administration (FDA) issued an Emergency Use Authorization for urgent use of the Pfizer-BioNTech COVID-19 vaccine (BNT162b2) for the prevention of COVID-19 for individuals aged 16 years and older. Then, the FDA approved the administration of BNT162b2 for individuals aged 12–15 years on May 10, 2021, and for individuals aged 5–11 years on October 29, 2021 [19]. Widespread vaccination across age groups is essential in ongoing efforts to curtail the pandemic [20]. Therefore, evaluating the adverse reactions of BNT162b2 vaccination in children and assessing whether this vaccine meets high safety standards is a crucial public health issue [21]. Previous studies have reported adverse reactions after BNT162b2 vaccination among individuals aged 5–11 years. Adverse reactions in this population were generally nonserious, more common after the second vaccination, and substantially less common than those observed among individuals aged 12–15 years [22–24]. Children with underlying comorbidities are at risk of severe COVID-19 [8; 25–29]; however, long-term data on the adverse reactions after BNT162b2 vaccination in these patients are limited.

Fukushima Prefecture, Japan, began administering BNT162b2 in two doses (10 μg, 0.2 mL each), 3 weeks apart to individuals aged 5–11 years on March 9, 2022. This prefecture has experienced a triple disaster: the radiation disasters, the Fukushima Daiichi Nuclear Power Plant accident, and the Great East Japan earthquake. Since these experiences, this prefecture has been an ongoing cross-sector collaboration among the local government, private and public medical sectors, and the community for over 10 years [30]. Based on these collaborations, this prefecture has been continuously subjected to COVID-19 antibody titer monitoring to develop infection prevention measures accordingly in the Fukushima Vaccination Community Survey (FVCS) [31–36]. This prefecture has some of the best information on antibody titers and adverse reactions after BNT162b2 vaccination in Japan, making it suitable for studying adverse reactions of BNT162b2 vaccination in the long-term based on the presence or absence of allergic diseases in individuals aged 5–11 years. Therefore, we aimed to investigate the type and frequency of adverse reactions in healthy and allergic disease individuals aged 5–11 years over the first 7 days following the first and second BNT162b2 vaccination.

## Materials and methods

### Study site, design, and participants

This was an observational and historical cohort study using a paper-based questionnaire from April 2, 2022, to June 29, 2022. The Seireikai group that runs Hirata Central Hospital is located in the Ishikawa district, which is a mountainous region and one of the most resource-poor areas in the Fukushima Prefecture. The Seireikai group and four municipalities (Hirata village, Tamagawa village, Ishikawa town, and Furudono town) in the Ishikawa district conducted a mass vaccination program for children together to secure physicians and manage adverse reactions specific to children. This vaccination program included sufficient time to explain the vaccine efficacy and safety to children and their parents/guardians, a free call center, follow-up on adverse reactions, and a questionnaire. The study population included children who had received the BNT162b2 vaccination at the Seireikai group during the mass vaccination programs. The eligibility criteria for the study participants consisted of individuals aged 5–11 years who had received two doses of the BNT162b2 vaccination (10 μg, 0.2 mL each) during the study period and consent from the children or their surrogates. Our results were compared with previously reported results for individuals aged 12–15 years [37].

### Questionnaire

We created a questionnaire consisting of two sections to study adverse reactions in healthy and allergic disease individuals aged 5–11 years throughout the 7 days following their first and second BNT162b2 vaccination. "Introduction" asked for the sociodemographic characteristics of the children, including their sex, age, height, weight, blood type, history of Bacille Calmette-Guerin (BCG) vaccination given as regular immunization services, allergic disease, medication, and history of COVID-19. "Materials and methods" asked for their adverse reactions 7 days following their first and second BNT162b2 vaccinations. This section assessed the presence of local pain, headache, diarrhea, dizziness, fatigue, muscle/joint pain, nausea, fever, swelling of BCG scar, worsening of chronic diseases (including asthma, hay fever, allergic rhinitis, atopic dermatitis, and food allergies), and medication use as adverse reactions. We designed these questions based on findings from experts and previous studies [31–38]. We enquired regarding the swelling of BCG scar following mRNA vaccination in the questionnaire since it has been reported in a previous study [38].

### Data collection

When the vaccination program for children was conducted, we distributed the questionnaire to the children or their surrogates and explained how to complete it. The parent or surrogate recorded their children's adverse reactions for 7 days following the first and second BNT162b2 vaccination and posted the results to the Seireikai group. The deadline for responses was June 29, 2022.

### Statistical analysis

We compared the participant characteristics using descriptive statistics according to the presence of allergic diseases. Allergic diseases included asthma, hay fever, allergic rhinitis, atopic dermatitis, and food allergies. We conducted a descriptive analysis of the characteristics of those with allergic diseases who experienced worsening of their chronic disease after the first and/or second BNT162b2 vaccination. Categorical variables (sex, blood type, BCG vaccination, allergic disease, medication, and COVID-19) are presented as frequencies, and continuous variables (age, height, weight, and body mass index [BMI)) are presented as the mean and standard deviation. We created a bar graph of the frequency of adverse reactions after the first and second BNT162b2 vaccinations. We also created a bar graph comparing the frequency of adverse reactions after the second dose of BNT162b2 vaccination among individuals aged 5–11 years and those aged 12–15 years [37]. Moreover, we conducted a chi-squared test for categorical variables and a t-test for continuous variables. Logistic multiple regression was used to assess the relationship between systemic adverse reactions that may have social consequences, such as school attendance, age, sex, BMI, history of BCG vaccination, asthma, hay fever, allergic rhinitis, atopic dermatitis, food allergies, and medication. Systemic adverse reactions were defined as any one of the following after the first or second BNT162b2 vaccination: headache, diarrhea, dizziness, fatigue, muscle/joint pain, nausea, fever, and medication used to treat an adverse reaction. We considered *p*-values < 0.05 to be statistically significant. The statistical software STATA/IC (version 15; Lightstone, DL, College Station, TX, USA) and Python (version 3.7.12) were used for all analyses.

## Results

### Participant characteristics

In four municipalities (Hirata village, Tamagawa village, Ishikawa town, and Furudono town) in the Ishikawa district, 1536 individuals aged 5–11 years were eligible for BNT162b2 vaccination, and 806 received two doses of the vaccination in the Seireikai group during the study period. Of the 806 individuals that were recruited, 421 (52.2%) consented to participate in the study. The mean age of the participants was 8.8 ± 1.9 years, the mean height was 132.8 ± 12.9 cm, 216 (51.3%) participants were male, 216 (51.3%) had allergic diseases, and 190 (45.1%) experienced systemic adverse reactions. Table 1 shows the allergic disease profile: 162 patients had hay fever, 71 had allergic rhinitis, 54 had atopic dermatitis, 46 had asthma, and 16 had food allergies. Of the 216 participants with allergic diseases, 45 (20.8%) experienced worsening of their chronic diseases after the first BNT162b2 vaccination, 41 (19.0%) experienced worsening of their chronic diseases after the second BNT162b2 vaccination, and 54 (12.8%) experienced worsening of their chronic diseases after the first and/or second BNT162b2 vaccination. The frequency of worsening of chronic diseases (*p* < 0.001), fatigue (*p* = 0.002), and nausea (*p* = 0.038) after the second BNT162b2 vaccination was significantly higher among individuals with allergic diseases.

**Table 1:** Participant characteristics based on the presence or absence of allergic disease (*n* = 421)

	Allergic disease (*n* = 216)	No allergic disease (*n* = 205)	Total (*n* = 421)	*P*-value
Male	112 (51.9)	104 (50.7)	216 (51.3)	0.82
Height (cm) (mean [SD])	134.1 [13.0]	131.4 [12.7]	132.8 [12.9]	0.053
Weight (kg) (mean [SD])	32 [8.7]	30.9 [9.9]	31.5 [9.3]	0.25
Age (mean [SD])	9 [1.9]	8.6 [1.9]	8.8 [1.9]	0.052
BMI^a^				
Below 25th percentile	39 (20.9)	43 (26.4)	82 (23.4)	0.22
Between 25–75th percentiles	82 (43.9)	62 (38.0)	144 (41.1)	0.23
Above 75th percentiles	66 (35.3)	58 (35.6)	124 (35.4)	0.96
Blood type				
A	57 (26.4)	48 (23.4)	105 (24.9)	0.48
B	25 (11.6)	30 (14.6)	55 (13.1)	0.35
O	41 (19.0)	39 (19.0)	80 (19.0)	0.99
AB	14 (6.5)	11 (5.4)	25 (5.9)	0.63
BCG vaccination	205 (94.9)	193 (94.1)	398 (94.5)	0.73
Allergic disease				
Asthma	46 (21.3)	0 (0.0)	46 (10.9)	-
Hay fever	162 (75)	0 (0.0)	162 (38.5)	-
Allergic rhinitis	71 (32.9)	0 (0.0)	71 (16.9)	-
Atopic dermatitis	54 (25.0)	0 (0.0)	54 (12.8)	-
Food allergies	16 (7.4)	0 (0.0)	16 (3.8)	-
Medication				
Steroid	4 (1.9)	3 (1.5)	7 (1.7)	0.76
Antihistamine	60 (27.8)	0 (0.0)	60 (14.3)	-
COVID-19	2 (0.9)	3 (1.5)	5 (1.2)	0.61
AR after first dose				
Local pain	172 (79.6)	155 (75.6)	327 (77.7)	0.32
Headache	26 (12.0)	20 (9.8)	46 (10.9)	0.45
Diarrhea	8 (3.7)	5 (2.4)	13 (3.1)	0.45
Dizziness	1 (0.5)	1 (0.5)	2 (0.5)	0.97
Fatigue	26 (12.0)	23 (11.2)	49 (11.6)	0.79
Muscle/joint pain	13 (6.0)	12 (5.9)	25 (5.9)	0.94
Nausea	2 (0.9)	3 (1.5)	5 (1.2)	0.61
Fever	15 (6.9)	17 (8.3)	32 (7.6)	0.60
Swelling of BCG scar	1 (0.5)	3 (1.5)	4 (1.0)	0.29
Others	7 (3.2)	6 (2.9)	13 (3.1)	0.85
Worsening of CD	45 (20.8)	6 (2.9)	51 (12.1)	< 0.001
Medication use	11 (5.1)	10 (4.9)	21 (5.0)	0.92
AR after second dose				
Local pain	158 (73.1)	153 (74.6)	311 (73.9)	0.73
Headache	30 (13.9)	30 (14.6)	60 (14.3)	0.83
Diarrhea	4 (1.9)	1 (0.5)	5 (1.2)	0.20
Dizziness	3 (1.4)	2 (1.0)	5 (1.2)	0.70
Fatigue	43 (19.9)	24 (11.7)	67 (15.9)	0.022
Muscle/joint pain	9 (4.2)	11 (5.4)	20 (4.8)	0.56
Nausea	11 (5.1)	3 (1.5)	14 (3.3)	0.038
Fever	26 (12)	19 (9.3)	45 (10.7)	0.36
Swelling of BCG scar	1 (0.5)	3 (1.5)	4 (1.0)	0.29
Others	7 (3.2)	7 (3.4)	14 (3.3)	0.92
Worsening of CD	41 (19)	5 (2.4)	46 (10.9)	< 0.001
Medication use	10 (4.6)	12 (5.9)	22 (5.2)	0.57
After 1st and/or 2nd dose				
Systematic AR^b^	98 (45.4)	92 (44.9)	190 (45.1)	0.92
Worsening of CD	48 (22.2)	6 (2.9)	54 (12.8)	< 0.001

Data are presented as means [standard deviation] or numbers (percentages) of participants

We conducted chi-squared tests for categorical variables and *t*-tests for continuous variables

*BMI* Body Mass Index, *AR* Adverse Reaction, *CD* Chronic Diseases, *BCG* Bacille Calmette-Guerin

^a^We used BMI percentiles for each age for Japanese children in 2000. Based on the respondents’ weight, height, sex, and age, the 25th percentile and below was defined as thin, the 25–75th percentile was defined as normal, and the 75th percentile and above was defined as overweight.

^b^According to “Pfizer-BioNTech COVID-19 Vaccine Reactions & Adverse Events” published by the Centers for Disease Control and Prevention, systemic adverse reactions include headache, diarrhea, dizziness, fatigue, muscle pain, nausea, fever, and medication use

### Time course of adverse reactions

**Fig. 1:**
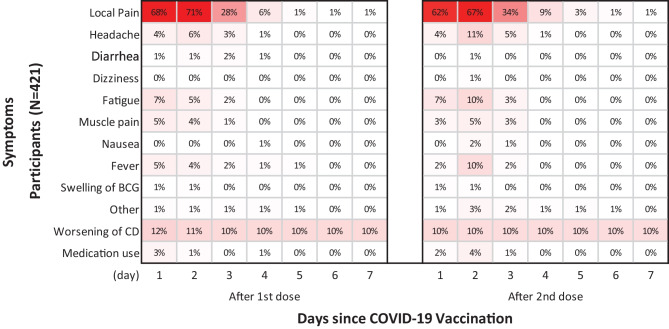
Time course of adverse reactions. The frequency of adverse reactions throughout 7 days following the first and second administration of BNT162b2 vaccination

**Fig. 2:**
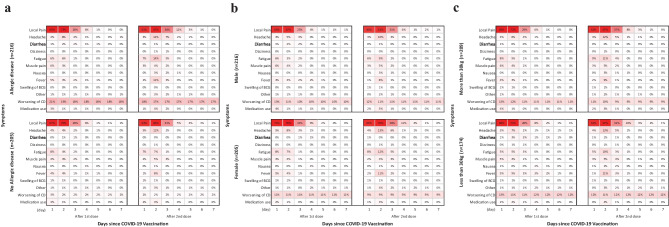
Time course of adverse reactions by **a** presence of allergic diseases, **b** sex, and **c** weight The frequency of adverse reactions throughout 7 days following the first and second administration of BNT162b2 vaccination according to participant demographics

For both the first and second BNT162b2 vaccination, local pain exhibited the highest frequency of approximately 70% 2 days after vaccination, and < 1% of cases continued for more than 6 days. No participants experienced systemic adverse reactions lasting longer than 5 days after both the first and second BNT162b2 vaccinations, except for fever in 1.0% of patients (Fig. 1). Of the 216 participants with allergic diseases, approximately 20% continued to experience worsening their chronic disease throughout the study period (Fig. 2a). No significant differences were observed in the time course of adverse reactions by sex or weight (Fig. 2b, c).

### Participants with allergic diseases who experienced worsening of their chronic diseases

Forty-eight (22.2%) of the 216 participants with allergic diseases, including 14 (30.4%) of the 46 participants with asthma, experienced worsening of their chronic diseases. The worsening of their chronic diseases lasted 5.9 ± 2.4 days after the first BNT162b2 vaccination and 5.4 ± 2.6 days after the second BNT162b2 vaccination (Supplementary Table 1). The mean age was significantly lower (*p* = 0.033), and the frequency of allergic rhinitis (*p* = 0.004), food allergy (*p* = 0.006), and antihistamine medication (*p* < 0.001) was significantly higher in the group with the allergic disease who experienced worsening of their chronic diseases. No significant difference was observed in the frequency of adverse reactions after the first and second BNT162b2 vaccination between groups with the allergic disease who experienced worsening of their chronic diseases and those who did not (Table 2).

**Table 2:** Characteristics of allergic diesease individuals according to worsening of chronic diseases after vaccination

	Worsening of CD (*n* = 48)	No worsening of CD (*n* = 168)	Total (*n* = 216)	*P*-value
Male	27 (56.3)	85 (50.6)	112 (51.9)	0.49
Height (cm) (mean [SD])	131.3 [13.1]	134.9 [12.9]	134.1 [13.0]	0.109
Weight (kg) (mean [SD])	29.7 [8.4]	32.7 [8.8]	32 [8.7]	0.053
Age (mean [SD])	8.4 [2.1]	9.1 [1.8]	9.0 [1.9]	0.033
BMI^a^				
Below 25th percentile	12 (28.6)	27 (18.6)	39 (20.9)	0.156
Between 25 and 75th percentiles	16 (38.1)	66 (45.5)	82 (43.9)	0.45
Above 75th percentile	14 (33.3)	52 (35.9)	66 (35.3)	0.81
Blood type				
A	13 (27.1)	44 (26.2)	57 (26.4)	0.90
B	8 (16.7)	17 (10.1)	25 (11.6)	0.21
O	5 (10.4)	36 (21.4)	41 (19.0)	0.086
AB	3 (6.3)	11 (6.5)	14 (6.5)	0.94
BCG vaccination	44 (91.7)	161 (95.8)	205 (94.9)	0.25
Allergic disease				
Asthma	14 (29.2)	32 (19.0)	46 (21.3)	0.131
Hay fever	38 (79.2)	124 (73.8)	162 (75.0)	0.45
Allergic rhinitis	24 (50.0)	47 (28.0)	71 (32.9)	0.004
Atopic dermatitis	12 (25.0)	42 (25.0)	54 (25.0)	1.00
Food allergies	8 (16.7)	8 (4.8)	16 (7.4)	0.006
Medication				
Steroid	1 (2.1)	3 (1.8)	4 (1.9)	0.89
Antihistamine	27 (56.3)	33 (19.6)	60 (27.8)	< 0.001
COVID-19 infection	0 (0.0)	2 (1.2)	2 (0.9)	0.45
AR after 1st dose				
Local pain	43 (89.6)	129 (76.8)	172 (79.6)	0.052
Headache	5 (10.4)	21 (12.5)	26 (12.0)	0.70
Diarrhea	2 (4.2)	6 (3.6)	8 (3.7)	0.85
Dizziness	0 (0.0)	1 (0.6)	1 (0.5)	0.59
Fatigue	4 (8.3)	22 (13.1)	26 (12.0)	0.37
Muscle/joint pain	1 (2.1)	12 (7.1)	13 (6.0)	0.194
Nausea	0 (0.0)	2 (1.2)	2 (0.9)	0.45
Fever	6 (12.5)	9 (5.4)	15 (6.9)	0.086
Swelling of BCG scar	1 (2.1)	0 (0.0)	1 (0.5)	0.061
Other	4 (8.3)	3 (1.8)	7 (3.2)	0.024
Medication use	2 (4.2)	9 (5.4)	11 (5.1)	0.74
AR after 2nd dose				
Local pain	39 (81.3)	119 (70.8)	158 (73.1)	0.151
Headache	3 (6.3)	27 (16.1)	30 (13.9)	0.083
Diarrhea	0 (0.0)	4 (2.4)	4 (1.9)	0.28
Dizziness	0 (0.0)	3 (1.8)	3 (1.4)	0.35
Fatigue	6 (12.5)	37 (22.0)	43 (19.9)	0.145
Muscle/joint pain	1 (2.1)	8 (4.8)	9 (4.2)	0.41
Nausea	2 (4.2)	9 (5.4)	11 (5.1)	0.74
Fever	5 (10.4)	21 (12.5)	26 (12)	0.70
Swelling of BCG scar	0 (0.0)	1 (0.6)	1 (0.5)	0.59
Other	2 (4.2)	5 (3.0)	7 (3.2)	0.68
AR After 1st and/or 2nd dose				
Medication use	2 (4.2)	8 (4.8)	10 (4.6)	0.86

Data are presented as means [standard deviation] or numbers (percentages) of participants

We conducted chi-squared tests for categorical variables and t-tests for continuous variables

*BMI* Body Mass Index, *AR* Adverse Reaction, *CD* Chronic Disease

^a^We used BMI percentiles for each age for Japanese children in 2000. Based on the respondents’ weight, height, sex, and age, the 25th percentile and below was defined as thin, the 25–75th percentile was defined as normal, and the 75th percentile and above was defined as overweight

### Comparison of frequency of adverse reactions

**Fig. 3:**
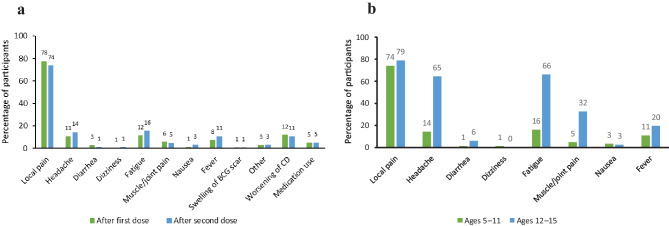
Bar graph comparing the frequency of adverse reactions **a** after the first and second administration of BNT162b2 vaccination for individuals aged 5–11 years and **b** after the second administration of BNT162b2 vaccination for individuals aged 5–11 years and 12–15 years

Comparing adverse reactions after the first vaccination with those after the second vaccination, the frequency of diarrhea was significantly higher after the second BNT162b2 vaccination, but no significant differences were observed in other adverse reactions (Fig. 3a) (Supplementary Table 2a). The frequency of headache (*p* < 0.001), diarrhea (*p* < 0.001), fatigue (*p* < 0.001), muscle/joint pain (*p* < 0.001), and fever (*p* < 0.001) after the second BNT162b2 vaccination was significantly lower in the individuals aged 5–11 years than in individuals aged 12–15 years (Fig. 3b) (Supplementary Table 2b).

### Factors associated with systemic adverse reactions

The results of multiple logistic regression analysis revealed that asthma (OR, 2.24; 95% CI, 1.08–4.66) was predominantly positively associated with experiencing systemic adverse reactions that could have social consequences, such as affecting school attendance. Age, sex, BMI, BCG vaccination history, allergic diseases other than asthma, and antihistamine medication were not significantly associated with experiencing systemic adverse reactions (Table 3).

**Table 3:** Logistic multiple regression analysis to identify variables that influence systemic ARs

	*B* (se)	OR (95% CI)	*P*-value
Age	0.060	1.01 (0.90–1.14)	0.84
Sex (base: male)	0.242	1.08 (0.70–1.68)	0.72
Obesity (base: normal)			
Thin	0.229	0.80 (0.46–1.40)	0.44
Overweight	0.174	0.69 (0.42–1.13)	0.142
BCG vaccination	0.835	1.45 (0.47–4.48)	0.52
Allergic diseases			
Asthma	0.837	2.24 (1.08–4.66)	**0.031**
Hay fever	0.224	0.91 (0.56–1.47)	0.69
Allergic rhinitis	0.292	0.94 (0.51–1.72)	0.83
Atopic dermatitis	0.313	0.85 (0.41–1.75)	0.66
Food allergies	0.798	1.27 (0.37–4.35)	0.70
Medication for CD			
Antihistamine	0.351	1.04 (0.53–2.01)	0.92

According to “Pfizer-BioNTech COVID-19 Vaccine Reactions & Adverse Events” published by the Centers for Disease Control and Prevention, systemic adverse reactions include headache, diarrhea, dizziness, fatigue, muscle pain, nausea, fever, and medication use

*B*(se) Partial regression coefficient, *OR* Odds ratio, *CI* Confidence Interval, *AR* Adverse Reaction, *CD* Chronic Disease

## Discussion

We studied individuals aged 5–11 years in the Ishikawa district to investigate the type and frequency of adverse reactions in healthy and allergic disease individuals aged 5–11 years over 7 days following the first and second BNT162b2 vaccination. This study revealed that among the patients with allergic diseases, 22.2% had experienced worsening of their chronic diseases, and the frequency of adverse reactions was higher than that of healthy individuals. Moreover, systemic adverse reactions were associated with asthma. Additionally, the frequency of adverse reactions was lower in those aged 5–11 years than in those aged 12–15 years. Fever was the only systemic adverse reaction that lasted longer than 5 days (1.0% of participants).

The type and frequency of adverse reactions after BNT162b2 vaccination differed according to the presence of allergic diseases. Moreover, 48 (22.2%) of the 216 participants with allergic diseases, including 14 (30.4%) of the 46 participants with asthma, experienced worsening of their chronic diseases. Individuals with allergic diseases rarely had systemic adverse reactions that lasted longer than 5 days after BNT162b2 vaccination, but they exhibited a higher frequency of fatigue and dizziness after the second BNT162b2 vaccination than healthy individuals. To our knowledge, this is the first report on the worsening of chronic diseases after BNT162b2 vaccination for children with asthma. However, similar observations after vaccination for other infectious diseases have been reported. For example, the worsening of asthma after inactivated influenza vaccination has been reported. The frequency was similar to that in the placebo group (33.6 and 33.0%, respectively) and was consistent with our results (30.4%) [39]. The higher frequency of adverse reactions in those with allergic diseases was consistent with previous reports on adults [40]. It has been suggested that individuals with allergic diseases, such as asthma, who are potentially susceptible to COVID-19 [8, 25–29], may experience more adverse reactions after BNT162b2 vaccination. Therefore, to ensure that children with allergic diseases receive the vaccine safely, further information regarding adverse reactions and long-term effects of BNT162b2 vaccination needs to be collected.

There were factors associated with the development of systemic adverse reactions. The results of multiple logistic regression analysis revealed that asthma (OR, 2.24; 95% CI, 1.08–4.66) was predominantly positively associated with experiencing systemic adverse reactions. In contrast, BMI was not associated with systemic adverse reactions in this study, which differed from previous reports of adults [41]. Risks for COVID-19 severity include being overweight and allergic diseases, including asthma [42]. Because asthma is also a risk factor for anaphylactic shock [43], monitoring systemic adverse reactions after BNT162b2 vaccination in children with asthma is vital.

The frequency of adverse reactions after BNT162b2 vaccination differed among those aged 5–11 years and those aged 12–15 years. Specifically, the frequency of headache (*p* < 0.001), diarrhea (*p* < 0.001), fatigue (*p* < 0.001), muscle/joint pain (*p* < 0.001), and fever (*p* < 0.001) after the second BNT162b2 vaccination was significantly lower in those aged 5–11 years than those aged 12–15 years. These results were consistent with previous reports [22, 23]. Moreover, no serious adverse reactions that would have required hospitalization occurred in individuals aged 5–11 years. In general, body weight is an important factor in clinical considerations of drug administration. Further investigation is warranted to determine how the three-fold difference in vaccine dosage between those aged 5–11 and those over 12 years of age (10 µg vs. 30 µg) and the difference in dosage per body weight affects safety after BNT162b2 vaccination.

Common adverse reactions after BNT162b2 vaccination did not last long. For both the first and second BNT162b2 vaccination, local pain had the highest frequency of about 70% 2 days after BNT162b2 vaccination, and less than 1% of cases continued for more than 6 days. Additionally, fever was the only systemic adverse reaction that lasted longer than 5 days after the first and second BNT162b2 vaccinations, occurring in 1.0% of cases. These results are consistent with previous reports that adverse reactions peaked at 1 or 2 days after BNT162b2 vaccination, and < 10% of those reactions lasted for 7 days [44]. Because the main reason people hesitate for vaccination is concern about adverse reactions [45], knowing that common adverse reactions for individuals aged 5–11 years end within 5 days are important.

Some limitations should be considered when interpreting the results of this study. First, it was not possible to assess whether the worsening of the chronic diseases was due to the administration of the BNT162b2 vaccine. To assess this, studies are needed to compare the frequency of worsening of chronic diseases in the BNT162b2 vaccination group with that in the placebo group. Second, the questionnaire response rate of 52.2% might affect the results and make generalization difficult because we were not able to examine the extent to which reporting bias might be present and the respondents were representative of the study’s population. Third, we could not clarify the level of side effects (e.g., the level of an asthma attack) since the self-administered questionnaire contains possible reporting bias. Fourth, due to the limited sample size, we could not detect rare adverse reactions following vaccination, such as anaphylactic shock. Despite these limitations, the present study was the first to examine adverse reactions in 27.4% of individuals aged 5–11 years in the Ishikawa district over a long period and investigate the factors that influence these reactions.

## Conclusion

In this study, we found that individuals aged 5–11 years with allergic diseases experienced worsening of their chronic diseases for 1 week after the first and second BNT162b2 vaccinations and had a higher frequency of commonly reported adverse reactions than healthy individuals. Individuals with allergic diseases, who are potentially susceptible to COVID-19, may experience more adverse reactions after BNT162b2 vaccination than healthy individuals. To ensure that children with allergic diseases receive the vaccine safely, further information needs to be collected regarding the adverse reactions and long-term effects of BNT162b2 vaccination and the mechanism causing these reactions.

## Supplementary Information

Below is the link to the electronic supplementary material.

Supplementary file1 (DOCX 34 KB)

## Data Availability

The data that support the findings of this study are available from the Seireikai Health Care Group; however, restrictions apply to the accessibility of these data, which were used under license for the current study, as they are not publicly available. Nevertheless, data are available from the authors upon reasonable request and with permission from the Seireikai Health Care Group.

## Abbreviations

BNT162b2: Pfizer-BioNTech COVID-19 vaccine
COVID-19: Coronavirus disease 2019
FDA: Food and Drug Administration
BMI: Body mass index
BCG: Bacille Calmette-Guerin
