# Supplementary material for: Time course of adverse reactions following BNT162b2 vaccination in healthy and allergic disease individuals aged 5–11 years and comparison with individuals aged 12–15 years: an observational and historical cohort study
Source: Eur J Pediatr. 2022 Oct 13;182(1):123–33. doi: 10.1007/s00431-022-04643-0 (PMC9556290; doi:10.1007/s00431-022-04643-0)
Supplement: Supplementary file 1 — Supplementary file1 (DOCX 34 KB) [file 431_2022_4643_MOESM1_ESM.docx]

Supplementary Table 1. Mean duration of adverse reactions (days) according to worsening of chronic diseases

|  | Worsening of CD (n = 48) | No Worsening of CD (n = 168) | Total (N = 216) | p-value |
| --- | --- | --- | --- | --- |
| Total days of AR after first dose (mean [SD]) |  |  |  |  |
| Local pain | 2.2 [1.0] | 1.7 [1.2] | 1.8 [1.2] | 0.055 |
| Headache | 0.1 [0.5] | 0.2 [0.6] | 0.2 [0.5] | 0.78 |
| Diarrhea | 0.1 [0.3] | 0.1 [0.4] | 0.1 [0.4] | 0.88 |
| Dizziness | 0.0 [0.0] | 0.0 [0.1] | 0.0 [0.1] | 0.59 |
| Fatigue | 0.1 [0.4] | 0.1 [0.4] | 0.1 [0.4] | 0.72 |
| Muscle/joint pain | 0.0 [0.0] | 0.1 [0.4] | 0.1 [0.4] | 0.156 |
| Nausea | 0.0 [0.0] | 0.0 [0.1] | 0.0 [0.1] | 0.45 |
| Fever | 0.1 [0.4] | 0.1 [0.6] | 0.1 [0.6] | 0.96 |
| Swelling of BCG scar | 0.1 [0.0] | 0.0 [0.0] | 0.0 [0.0] | n/a |
| Other | 0.1 [0.4] | 0.0 [0.4] | 0.1 [0.4] | 0.38 |
| Worsening of CD | 5.9 [2.4] | 0.0 [0.0] | 1.3 [2.7] | <0.001 |
| Medication use | 0.1 [0.3] | 0.1 [0.3] | 0.1 [0.3] | 0.85 |
| Total days of AR after second dose (mean [SD]) |  |  |  |  |
| Local pain | 2.1 [1.4] | 1.7 [1.4] | 1.8 [1.4] | 0.103 |
| Headache | 0.1 [0.6] | 0.3 [0.8] | 0.2 [0.8] | 0.27 |
| Diarrhea | 0.0 [0.0] | 0.0 [0.2] | 0.0 [0.2] | 0.31 |
| Dizziness | 0.0 [0.0] | 0.0 [0.2] | 0.0 [0.2] | 0.38 |
| Fatigue | 0.1 [0.4] | 0.3 [0.7] | 0.3 [0.6] | 0.109 |
| Muscle/joint pain | 0.0 [0.2] | 0.1 [0.4] | 0.1 [0.3] | 0.52 |
| Nausea | 0.1 [0.3] | 0.1 [0.5] | 0.1 [0.5] | 0.84 |
| Fever | 0.1 [0.4] | 0.2 [0.5] | 0.2 [0.5] | 0.58 |
| Swelling of BCG scar | 0.0 [0.0] | 0.0 [0.2] | 0.0 [0.1] | 0.59 |
| Other | 0.2 [1.1] | 0.1 [0.1] | 0.1 [0.5] | 0.021 |
| Worsening of CD | 5.4 [2.6] | 0.0 [0.0] | 1.2 [2.7] | <0.001 |
| Medication use | 0.0 [0.2] | 0.1 [0.4] | 0.1 [0.3] | 0.64 |

Data are presented as means [standard deviation]. Abbreviations: AR, adverse reaction; CD, chronic disease; BCG, Bacille Calmette-Guerin. We conducted t-tests for continuous variables.

Supplementary Table 2a. Frequency of adverse reactions after the first and second vaccination

|  | After first dose | After second dose | p-value |
| --- | --- | --- | --- |
| Local pain | 327 (77.7) | 311 (73.9) | 0.198 |
| Headache | 46 (10.9) | 60 (14.3) | 0.146 |
| Diarrhea | 13 (3.1) | 5 (1.2) | 0.057 |
| Dizziness | 2 (0.5) | 5 (1.2) | 0.25 |
| Fatigue | 49 (11.6) | 67 (15.9) | 0.072 |
| Muscle/joint pain | 25 (5.9) | 20 (4.8) | 0.44 |
| Nausea | 5 (1.2) | 14 (3.3) | 0.037 |
| Fever | 32 (7.6) | 45 (10.7) | 0.12 |
| Swelling of BCG scar | 4 (1.0) | 4 (1.0) | 1.00 |
| Other | 13 (3.1) | 14 (3.3) | 0.84 |
| Worsening of CD | 51 (12.1) | 46 (10.9) | 0.59 |
| Medication use | 21 (5.0) | 22 (5.2) | 0.88 |

Data are presented as numbers (percentages) of participants. Abbreviations: AR, adverse reaction; CD, chronic disease; BCG, Bacille Calmette-Guerin. We conducted chi-squared tests for categorical variables.

Supplementary Table 2b. Frequency of adverse reactions after the second vaccination between ages 5–11 and 12–15

|  | Ages 5–11 (n = 421) | Ages 12–15 (n = 1097) | p-value |
| --- | --- | --- | --- |
| Local pain | 311 (73.9) | 866 (78.9) | 0.22 |
| Headache | 60 (14.3) | 708 (64.5) | <0.001 |
| Diarrhea | 5 (1.2) | 65 (5.9) | <0.001 |
| Dizziness | 5 (1.2) | n/a | n/a |
| Fatigue | 67 (15.9) | 726 (66.2) | <0.001 |
| Muscle/joint pain | 20 (4.8) | 335 (32.4) | <0.001 |
| Nausea | 14 (3.3) | 29 (2.6) | 0.42 |
| Fever | 45 (10.7) | 215 (19.6) | <0.001 |

Data are presented as numbers (percentages) of participants. We conducted a chi-squared test for categorical variables.

Supplementary Table 3. Participant characteristics according to presence or absence of systemic adverse reactions after vaccination

|  | Systemic AR^1^ (n = 190) | Non-Systemic AR (n = 231) | p-value |
| --- | --- | --- | --- |
| Male | 98 (51.6) | 118 (51.1) | 0.92 |
| Height (cm) (mean [SD]) | 132.9 [13.2] | 132.6 [12.8] | 0.70 |
| Weight (kg) (mean [SD]) | 30.8 [8.8] | 31.5 [9.7] | 0.68 |
| Age (mean [SD]) | 8.8 [1.9] | 8.7 [1.9] | 0.93 |
| BMI ^2^ |  |  |  |
| Below 25th percentile | 37 (23.6) | 45 (23.3) | 0.96 |
| Between 25–75th percentiles | 71 (45.2) | 73 (37.8) | 0.097 |
| Above 75th percentile | 49 (31.2) | 75 (38.9) | 0.20 |
| Blood type |  |  |  |
| A | 49 (25.8) | 56 (24.2) | 0.72 |
| B | 27 (14.2) | 28 (12.1) | 0.53 |
| O | 32 (16.8) | 48 (20.8) | 0.31 |
| AB | 14 (7.4) | 11 (4.8) | 0.26 |
| BCG vaccination | 182 (95.8) | 216 (93.5) | 0.31 |
| CD | 98 (51.6) | 118 (51.1) | 0.92 |
| Type of CD |  |  |  |
| Asthma | 27 (14.2) | 19 (8.2) | 0.050 |
| Hay Fever | 74 (38.9) | 88 (38.1) | 0.86 |
| Allergic rhinitis | 35 (18.4) | 36 (15.6) | 0.44 |
| Atopic dermatitis | 26 (13.7) | 28 (12.1) | 0.63 |
| Food allergies | 8 (4.2) | 8 (3.5) | 0.69 |
| Other ^3^ | 22 (11.6) | 18 (7.8) | 0.125 |
| Medication | 4 (2.1) | 3 (1.3) | 0.52 |
| Steroid | 4 (2.1) | 3 (1.3) | 0.52 |
| Antihistamine | 29 (15.3) | 31 (13.4) | 0.59 |
| COVID-19 | 4 (2.1) | 1 (0.4) | 0.115 |

Data are presented as means [standard deviation] or numbers (percentages) of participants. Abbreviations: AR, adverse reaction; CD, chronic disease. We conducted chi-squared tests for categorical variables and t-tests for continuous variables.

^1^ Systemic adverse reactions include headache, diarrhea, dizziness, fatigue, muscle pain, nausea, fever and medication use.

^2^ We used BMI percentiles of each age for Japanese children in 2000. Based on the respondents' weight, height, sex and age, the 25th percentile and below was defined as thin, the 25–75th percentile as normal, and the 75th percentile and above as overweight.

^3^ Other chronic diseases included cleft palate, atrial septal defect, ventricular septal defect, tetralogy of Fallot, total anomalous pulmonary venous return, constipation, otitis media, genetically-determined epidermolysis bullosa, cardiac tumor, chronic subdural hematoma, autism spectrum disorder, attention-deficit/hyperactivity disorder, west syndrome, disorders of autonomic nervous system, hydrocephalus, transient congenital hypothyroidism, and type 1 diabetes mellitus.

Supplementary Table 4 Logistic regression analysis excluding medication from the definition of systemic ARs to identify variables that influence systemic ARs.

|  | B (se) | OR (95% CI) | p-value |
| --- | --- | --- | --- |
| Age | 0.060 | 1.01 (0.90–1.14) | 0.82 |
| Sex (base: male) | 0.235 | 1.05 (0.68–1.63) | 0.82 |
| Obesity (base: normal) |  |  |  |
| Thin | 0.230 | 0.81 (0.46–1.40) | 0.45 |
| Overweight | 0.169 | 0.67 (0.41–1.09) | 0.109 |
| BCG vaccination | 0.828 | 1.45 (0.47–4.48) | 0.52 |
| Allergic diseases |  |  |  |
| Asthma | 0.835 | 2.23 (1.07–4.65) | **0.031** |
| Hay fever | 0.227 | 0.92 (0.57–1.50) | 0.74 |
| Allergic rhinitis | 0.293 | 0.94 (0.51–1.73) | 0.85 |
| Atopic dermatitis | 0.316 | 0.86 (0.42–1.77) | 0.68 |
| Food allergies | 0.802 | 1.28 (0.37–4.37) | 0.70 |
| Medication for CD |  |  |  |
| Antihistamine | 0.351 | 1.03 (0.12–2.61) | 0.47 |

Abbreviations: B (se), partial regression coefficient; OR, odds ratio; CI, confidence interval; AR, adverse reaction; CD, chronic disease.

Systemic adverse reactions include headache, diarrhea, dizziness, fatigue, muscle pain, nausea, and fever.
